# Embryonal life histories: Desiccation plasticity and diapause in the Argentinean pearlfish *Austrolebias bellottii*

**DOI:** 10.1002/ece3.4599

**Published:** 2018-10-30

**Authors:** Tom J. M. Van Dooren, Irma Varela‐Lasheras

**Affiliations:** ^1^ Centre for Biodiversity Naturalis Leiden The Netherlands; ^2^ CNRS/UPMC/UPEC/UPD/IRD/INRA – UMR 7618, Institute for Ecological and Environmental Sciences Paris (iEES) Sorbonne University Paris France

**Keywords:** desiccation plasticity, diapause, eco‐evo‐devo, embryonal life histories, mixed effect Cox models, multistate prediction, structured populations

## Abstract

Embryos of annual killifish diapause in soil egg banks while ponds are dry. Their rates of development and survival in different developmental stages determine the numbers and stages of embryos at rewetting. In the Argentinean pearlfish *Austrolebias bellottii*, we investigated plasticity for desiccation in such embryonal life history components across phases of mild desiccation and rewetting and also effects of life history on hatching. In comparison with nonannuals, our data suggest that incidences of diapause have become relatively independent of the occurrence of desiccation, as if they have become genetically assimilated. We found limited survival effects of desiccation, limited developmental delays, and an acceleration of development into the prehatching stage. This response can be adaptive when desiccation informs that an opportunity to hatch approaches. Embryos arrest development in the prehatching stage (diapause DIII) or in the dispersed‐cell phase (diapause DI). Parental pair variation in rates of development and survival in the earliest developmental stages affects the fraction of embryos that are in DI at rewetting and the number surviving. Given such effects on life history fitness components, rates during embryonal development seem "visible" to selection and the developmental system can thus adapt when pair variation contains a heritable component. In agreement with expectations for the presence of diversified bet‐hedging, some embryos hatched and others not in over half of the clutches with several developed embryos at the moment of rewetting. Hatching probabilities increased for eggs produced later in the experiment, and they increased when embryos were rewetted a second time after two months. This response is opposite of what is expected when age‐dependent hatching would be adapted to exploit opportunities for completing another generation before the dry season.

## 1 INTRODUCTION

Diapause, where the progress of development is interrupted or halted, is an emblematic trait in the study of evolution in variable environments. It is used to explain that evolution can favor delaying strategies and bet‐hedging (Evans & Dennehy, 2005; Philippi & Seger, 1989; Stearns, 2000). The adaptive target phenotype can be a distribution of individuals over states (remain in diapause/exit from it), and life history processes that affect the numbers of individuals in different states at a given point in time are essential to understand adaptation. Individual variability in diapause or other delaying strategies can have components of bet‐hedging and (transgenerational) plasticity together with genetic variation (Haccou & Iwasa, 1995; Van Dooren & Brendonck, 1998; Furness, Lee, & Reznick, 2015; Polačik et al., 2017). This complexity partly follows from its defining characteristics: diapause is not an instantaneous, easily reversible response to adversity (Danks, 1987), but a developmental and metabolic arrest that can also occur in the absence of adverse environmental conditions or before they arrive. This differentiates diapause from other types of developmental arrest such as quiescence (which only happens in the presence of adverse environmental conditions) or delayed hatching (which does not encompass a metabolic arrest and is eventually deleterious for the embryo, Darken, Martin, & Fisher, 1998).

The study of individual developmental life histories where diapause can occur presents an excellent opportunity to link proximate drivers and properties of developmental processes with demography and adaptation (Childs, Metcalf, & Rees, 2010). Attempts have been made to even link the presence of diapause to phylogenetic evolutionary processes of species selection (Helmstetter et al., 2016). However, the analysis of diapause in real‐world systems is often more intricate than in the canonical example of desert annuals with a seed bank (Childs et al., 2010), which could be one of the reasons that evidence for bet‐hedging for example often remains limited to a demonstration of the presence of individual variation compatible with it (Simons, 2011). Similarly, definitions of adaptive developmental plasticity (Nettle & Bateson, 2015) tend to focus on the reliability of available information in the environment for predicting which target phenotype should be constructed. However, individual stochastic variability in developmental processes can be a component of adaptation too, leading to plastic adaptive target distributions of phenotypes. Moreover, if survival varies between developmental stages, the integrated developmental system should account for expected survival when adapting to achieve a particular distribution of individual phenotypic states.

**Figure 1:**
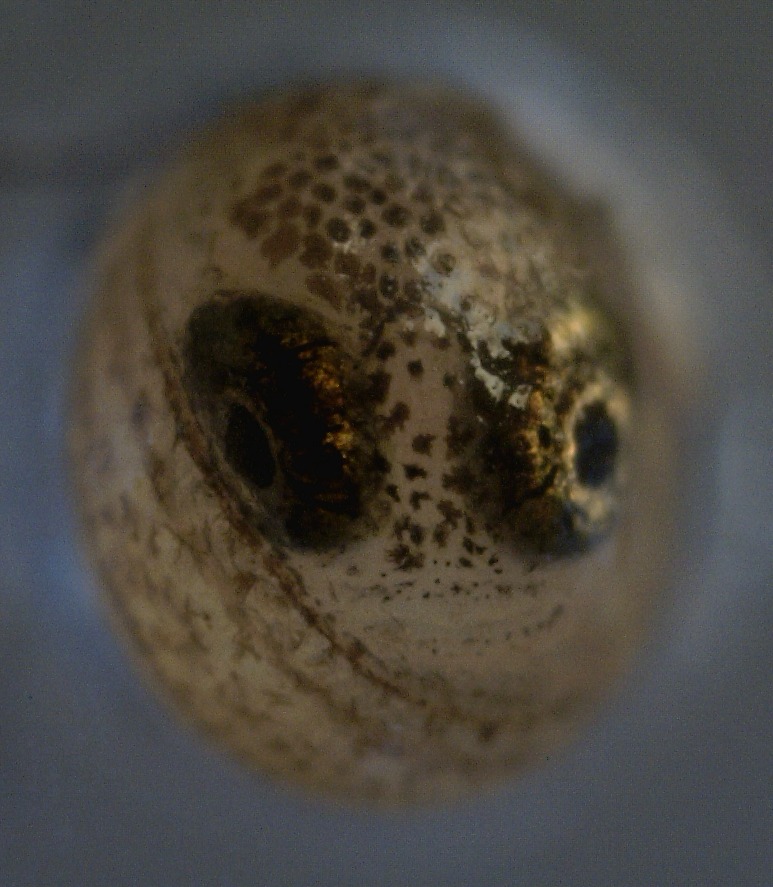
An *Austrolebias* annual killifish embryo in the pre‐hatching stage (diapause III)

Annual killifish are oviparous cyprinodontiform fish species from the suborder Aplocheiloidei (Berois, García, & Sá, 2015) receiving increasing attention due to their potential as model organisms. Annual fish use diapause to persist in temporary ponds of Africa and South America that dry out seasonally. Diapausing embryos buried in the soil survive and can hatch when the opportunity presents itself. This "annualism" life history has evolved repeatedly (Furness, Reznick, Springer, & Meredith, 2015; Helmstetter et al., 2016). Diapause in annual killifish can occur in three developmental stages (Wourms, 1972a, 1972c): Diapause I takes place in a unique developmental stage between epiboly and the onset of embryogenesis where the blastomeres individually disperse. Diapause II in the long‐somite embryo can coincide with a delay in the development of anterior structures relative to the rest of the body (Furness, Reznick, et al., 2015; Podrabsky, Garrett, & Kohl, 2010). Diapause III occurs when the embryo is completely developed and ready to hatch (Figure 1). As these diapauses can occur in three developmental stages, estimating the contributions of phenotypic plasticity and bet‐hedging to embryonal life history variation requires a structured approach. Among annual species studied thusfar, the presence of diapause I, II, and III and the environmental cues that affect their onset, duration, and termination seem variable (Wourms, 1972a, 1972b, 1972c; Markofsky & Matias, 1977; Markofsky, Matias, Inglima, Vogelman, & Orentreich, 1979; Inglima, Perlmutter, & Markofsky, 1981; Matias and Markofsky, 1978; Levels & Denuce, 1988).

Furness, Lee, et al. (2015) have demonstrated contributions of phenotypic plasticity to the entrance into diapause and a pattern consistent with bet‐hedging in hatching responses. Polačik et al. (2017) similarly found variability in developmental duration compatible with the presence of bet‐hedging and demonstrated that the largest component of developmental variation might be of maternal origin. However, Furness, Lee, et al. (2015) defined developmental duration as time to hatching or death, conflating development, and survival. Polačik et al. (2017) censored individuals that died in the experiment, but did not analyze effects of survival variability on developmental outcomes. To explain a decreased incidence of diapause in warmer temperatures, Furness, Lee, et al. (2015) proposed that temperature predicts the probability that another generation can be completed before the cool dry season starts. Similarly, Polačik et al. (2017) proposed that eggs from younger mothers develop faster to exploit an extra opportunity to hatch and reproduce within a season. An alternative explanation could be that developing fast is the best option given expected survival rates in the different stages.

Wourms (1972c) and Varela‐Lasheras and Van Dooren (2014) pointed out a different aspect of the evolution of annualism, by suggesting that the diapauses in annual killifish might have originated in a scenario of genetic assimilation (Waddington, 1942). This has become a mainstream evolutionary concept since Lande (2009) demonstrated by means of a standard quantitative genetic model that plasticity evolution can induce a process of genetic assimilation when environments shift abruptly. Varela‐Lasheras and Van Dooren (2014) showed that nonannual rivulid killifish slow down early development in response to a brief period of mild desiccation, that stage‐specific survival effects occur and that hatching can be delayed over a prolonged period. Their plastic hatching delay showed several characteristics of diapause III which is constitutive in annuals. Since then, Furness, Reznick, Tatarenkov, and Avise (2018) showed that developmental properties presumed to be limited to annuals do occur in some sister taxa. For annual killifish, there are no data allowing a detailed analysis of rates of development and survival during and after a desiccation period. We have no embryonal life history that we can use to assess fitness effects and optimal strategies, and we are therefore not approaching the use of modeling tools advocated by Childs et al. (2010).

Here, we collect demographic data and model structured embryonal life histories, while we impose desiccation instead of cues such as temperature which are associated with seasonal change but also with short‐term (even diurnal) fluctuations. Wourms (1972c) contains a brief statement that eggs of the Argentinean pearlfish *Austrolebias bellottii* (Steindachner, 1881), an annual killifish from the sister taxon of nonannual rivulids, enter diapause I and II more often when subjected to partial desiccation, but data were not presented. An explanation for this response could be that desiccation informs that the dry season has started; hence, there is no further opportunity to complete a generation within the same year.

Therefore, to investigate how a potentially adaptive distribution of phenotypes is achieved, we exposed embryos of the Argentinean pearl killifish *Austrolebias bellottii* in different stages of development, at different ages and with parents of different ages, to a substantial period of mild desiccation. We analyzed the effects on stage‐specific survival and developmental rates and on diapause incidence. To improve our understanding of how selection might operate on rates of survival and development, we determined how fitness components such as total number of embryos and the fraction in diapause I depend on these underlying rates. Embryos were rewetted so that variation in hatching probability and lagged effects on the further life histories of unhatched embryos could be assessed. We checked whether components of individual variation are compatible with *bet‐hedging*, whether developmental and hatching patterns could involve an adaptation to the potential of completing a *second generation* within the same year and whether desiccation is used as a reliable cue for the start of the *dry season*.

## 2 METHODS

### 2.1 Housing and data collection

Animal care and handling protocols were approved by the Animal Welfare committee of the Leiden Faculty of Sciences and Medicine. The twenty‐eight individuals of *Austrolebias bellottii* used in this experiment (14 males and 14 females from different family lines) were descendants from a population of 50 individuals collected in Ingeniero Maschwitz, Argentina, in 2004 and hatched from eggs collected in 2008. There were two groups of pairs with an age difference of about 4 months (hatched 2 or 6 months before the start of egg collection, on April 20, 2010). Pairs were kept in 20‐L aquaria in a climate room at 19°C with a 12‐hr light/dark cycle. The aquaria contained some plants (e.g. *Elodea densa*,* Vesicularia dubyana*,* Lemna minor*) and a black, plastic 2‐L container with boiled coco peat where the fish could hide and lay eggs.

**Figure 2:**
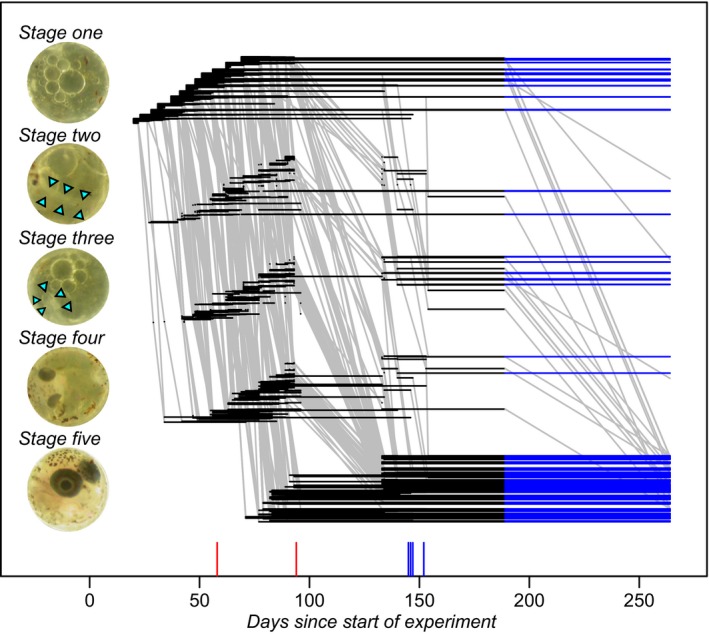
An overview of the "raw" data on embryonal life histories. Embryos were classified into five developmental stages as explained in the text, or as dead (this absorbing state is not represented). Per egg and stage, a horizontal line is drawn between the days of the first and last observation in that stage. Eggs are ordered within stage according their collection day. A blue line is drawn for eggs that were observed in the same stage at second rewetting (on day 264) as at the end of the period where regular observations were made (day 189). Developmental transitions are drawn as gray lines. They connect the last observation in a stage with the first observation in the next stage where the egg was observed. Note that no observations were made between days 96 and 133. In the insets depicting the different developmental stages, small triangles indicate the location of the embryo when not easily visible (stage 2: somites and notochord; stage 3: head region)

We collected eggs on different days, with intervals of 3–7 days between them. The day before collecting eggs, a clean container with 300 ml glass beads and 300 ml peat granules (both sieved and boiled) replaced the other container (Moshgani & Van Dooren, 2011). Twenty‐four hours later, eggs were collected and placed individually in wells of a 24‐well plate with 1 ml of UV‐sterilized tap water (DUNEA Leiden, the Netherlands). Each clutch was divided over two plates placed in different climate rooms with a small temperature difference between them (19.5°C vs. 20.5°C). Temperature differences between eggs and with adults were unplanned and due to within‐ and between room variability. Next to the control treatment, which consisted of eggs continually incubated in water, we exposed the eggs to different “desiccation” treatments in the range where expected survival of most stages would allow developmental responses to be observed (Podrabsky, Carpenter, & Hand, 2001). We set up desiccators with demineralized water and saturated salt solutions of KNO_3_, NH_4_H_2_PO_4_ and KCl that would generate air humidities from 100% relative humidity (Water), 94%–93%, 93% and 86%, respectively (O'Brien, 1948). The humidity levels were verified with dataloggers (Gemini Tinytag PlusII, https://www.geminidataloggers.com) which could only confirm that the relative humidity was below 100% for KCl (relative humidity 85%). Loggers might have failed to capture humidities reliably due to wear on the probe or limited precision at high humidities, or some solutions were insufficiently saturated and did not produce the expected regime (i.e., remained above 93%). We therefore decided to analyze differences between the different desiccation regimes with categorical variables and not by means of regressions. Developmental states were determined at least once per week for each individual embryo, except for a single longer interval in summer (Figure 2). Eggs were transferred to the desiccation treatments at ages between 17 and 50 days. Desiccation treatments lasted between 51 and 94 days. Treatments and control were terminated by replacing the water in the wells with a mix of tap water, demineralized water and peat extract to promote hatching (Varela‐Lasheras & Van Dooren, 2014). Embryos were checked for hatching responses over successive days, and hatchlings were removed from their well on the same day. Approximately 30 days later, the water was replaced with sterilized water again for the eggs that had not hatched. There were no further observations made during a period of 61 days. The hatching procedure was then repeated (112–119 days after the first rewetting). In all, embryos experienced environmental sequences with up to four changes and were followed for up to 244 days.

Based on previous studies (Varela‐Lasheras & Van Dooren, 2014; Wourms, 1972a), individual state was scored as "dead" or as being alive in one of five developmental stages that can be distinguished well (Figure 2). Embryos were assigned to stage 1 when collected. A neural keel and the first somites appearing delineate the start of stage 2, the presence of optic cups the transition to stage 3 and the pigmentation of the eyes the transition to stage 4. Finally, stage 5 starts when the embryo completely surrounds the yolk sac and ends when the fry hatches or dies. For embryos in stages four and five, we noted at each observation whether the tail coiled over the left or right side of the head. Diapause I occurs in the first stage, diapause II near the end of stage 2 or early in stage 3, diapause III in stage 5.

### 2.2 Statistical analysis

Developmental life histories consist of the age intervals embryos spend in each developmental stage before either moving into the next stage, hatching, or dying (Figure 2). Events (deaths, developmental transitions) are assumed to have occurred just before they are observed. The data were analyzed using Cox proportional hazard models with random effects, using the libraries survival and coxme in R (Therneau, 2015; Therneau & Grambsch, 2000). We modeled all transition rates per stage separately, that is, the mortality rates and developmental transitions to the next stage (Varela‐Lasheras & Van Dooren, 2014). The models per stage in the main text combine all durations in the different environmental conditions, so that effects of environmental conditions can be tested by removing them from a model. In the supplement, results are presented for models where the durations were analyzed per environment separately (Control/Desiccation Treatments/Rewetted) or censored before the long interobservation summer interval. In some environment/stage combinations, we had very few individuals at risk and therefore either did not fit any models to the data, or Cox models with fixed effects. We analyzed zero‐one responses or proportions of states present at specific time points using logistic regressions.

#### 2.2.1 Survival

Per stage, we first fitted a Cox model with period (control "Wet"/desiccation "Dry"/"Rewetted") and salt treatment (categories "H_2_O"/"KNO_3_"/"NH_4_H_2_PO_4_"/"KCl") fixed effects, their interactions and temperature and parental age (categorical, "Young"/"Old") fixed effects. Temperature treatments are unreplicated and can therefore not be separated from a room effect. For stages two to five, we added the duration spent in the preceding stage as a covariate. The model contained a parental pair random effect, a random effect of collection date and a plate effect. This model was then simplified by removing effects that were not significant in *χ* ^2^ likelihood ratio tests. We sequentially tested and deleted nonsignificant effects in a fixed order: first the tests for pair variation, collection date, plate variation; then previous duration effects, salt treatments, the period effect, temperature and parental age effects. The models in the Supplement underwent the same model selection procedure. When an effect was significant in a likelihood ratio test but the parameter estimate had a very large standard error or there were data lacking to estimate a subset of parameters, we also removed that effect from the model or analyzed a subset of the data. We replaced random collection date effects by a fixed linear effect to test whether mothers produced eggs with different characteristics early and late in the experiment.

#### 2.2.2 Development

Age within stage is used as the appropriate time scale to investigate rates of development. The data were analyzed starting from the same maximal models as for survival and we tested effects in the same order. Spontaneous hatching from stage 5 was modeled as a developmental transition, while hatching induced by rewetting caused censoring.

#### 2.2.3 Sensitivities of fitness components to rates

In any developmental stage, development and death are risks that are competing. Inspecting risks of a separate process is insufficient to estimate distributions of individuals over states at different points in time. We used multistate estimation of fractions per stage and made plots of these fractions as a function of age using the mstate library for R (de Wreede, Fiocco, & Putter, 2011). These fractions were used to assess the importance of each separate survival rate and rate of development on two fitness components: (a) total number of surviving embryos at the end of the observation period and (b) the fraction in diapause I. The data for the control group were bootstrapped (100 resamples) and multistate estimation was carried out for each pseudo‐dataset. The estimated cumulative hazard for each specific rate was increased and decreased by multiplication with 11 equally spaced values between 0.9 and 1.1. The effect of this rescaling on the two fitness components was calculated and the averages of the resulting lines across bootstrap resamples plotted on graphs. The slopes of the lines can be interpreted as local slopes of phenotype landscapes (Rice, 2004) or as sensitivities. This analysis allows us to state which changes in rates lead to a change in the fraction of slow developing eggs, which is key to understand whether rates are adapted to the possibility of completing another generation within the same year.

#### 2.2.4 Fraction in diapause

We investigated whether the distribution of embryos across stages at the moment of rewetting and at the end of the experiment depended on previous individual history. We analyzed the data with a set of binary logit models, each for the fraction of embryos in an earlier developmental stage relative to the number in stage 5 (baseline category logit generalized linear models (glm); Agresti, 2013). Mixed binary models did not converge well here. We therefore restricted the analysis to fixed effects of desiccation treatment, time spent in the treatment and their interaction, individual age, parental pair, collection date, and temperature effects. We used the inverse of the time spent in a treatment as an explanatory variable such that intercepts per treatment became the expectation for when embryos would have been in the treatment for an infinite amount of time. Model selection was carried out using likelihood ratio tests. When the desiccation treatment × time interaction was removed, we also removed time in treatment from the model, as it was then confounded with individual age. For survival and development rates where we found significant parental variation, we tested whether parental pair effects could be simplified by only considering parental age. We estimated Spearman's rank correlations of the random parental effects with the total number of surviving embryos and the fraction in diapause I at rewetting.

#### 2.2.5 Coiling

For embryos in stage 4 and 5, we repeatedly observed the direction in which they coiled their tail over the head, and used the rate of change in direction as a proxy for activity (Varela‐Lasheras & Van Dooren, 2014). We estimated how the probability of a transition depended on treatment, parental pair, and time spent in stages four or five. We used glm's for the occurrence of a change in coiling (positive response) and with a complementary log‐log link, so that by means of an offset, we could correct for the duration between observations (Harney, Van Dooren, Paterson, & Plaistow, 2013).

#### 2.2.6 Hatching

The probabilities of hatching when water with peat extract was added are reported. We analyzed these probabilities of hatching using binary logit glm's. Individuals that hatched within three days of adding peat water were scored as a positive response, the ones that remained in stage 5 as nonhatchers. Individuals that were found dead in this time interval were not included in the analysis. This model contained effects of temperature, collection date, date where hatching water was added, desiccation treatment, parental pair, time spent in stage 5, time spent in treatment and interactions of desiccation treatment with the two time variables. Model selection was carried out using likelihood ratio tests.

## 3 RESULTS

We collected and incubated 2,161 eggs in total (12 collection days). A total of 539 embryos were subjected to desiccation treatments and 599 embryos were rewetted, 360 of them twice. The median number of observations per embryo was 16 and the maximum 31. The desiccator with KCl contained fewer embryos at risk than the other treatments (Table 1). We did that to minimize individual losses at the strongest level of desiccation.

**Table 1:** Number of individuals at risk (number of developmental transition events, number of deaths within stage) per stage, per period and per salt used for desiccation

Stage	Wet	Desiccated				Rewetted
		KCl	KNO3	NH4H2PO4	H2O	
1	2,161 (595/1,091)	8 (4/2)	62 (43/15)	48 (34/11)	101 (75/15)	42 (2/3)
2	535 (410/18)	6 (4/2)	57 (49/7)	54 (44/5)	111 (103/6)	13 (7/1)
3	422 (273/13)	9 (7/0)	91 (84/6)	77 (74/3)	145 (134/7)	21 (2/0)
4	242 (126/10)	21 (18/0)	107 (93/12)	96 (84/8)	154 (139/14)	15 (8/2)
5	199 (8/0)	28 (0/1)	111 (0/20)	106 (1/21)	162 (2/4)	536 (0/58)

### 3.1 Survival

**Figure 3:**
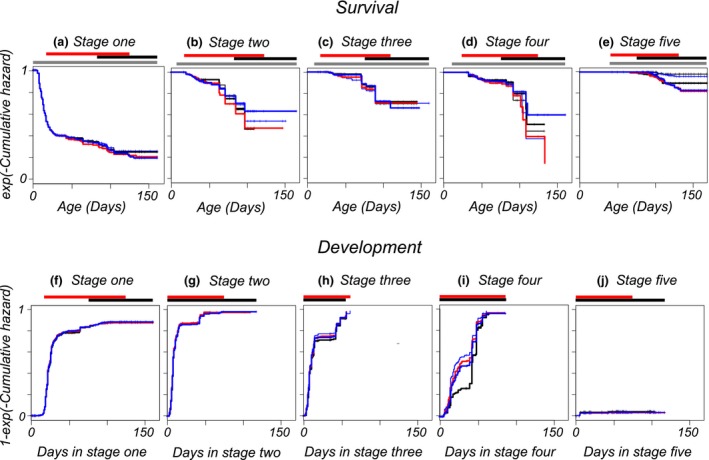
Rates of death and development compared by means of transformed cumulative hazard functions. Each panel contains the data for a specific developmental stage and has a separate curve per treatment group (black, thin: control group; blue, thin: desiccator with H_2_0; red thick: KNO_3_; blue thick: NH_4_H_2_PO_4_; black, thick: KCl). At many instances, embryos within the same stage were distributed over different environmental regimes (control/dry/rewetted). Bars above panels indicate ages (in days) whether any embryos were in that stage (gray, survival), in the desiccation regime (red) or rewetted (black). The curves in panels representing survival are of cumulative hazards *S*, estimated using Fleming‐Harrington estimators and transformed according *exp*(‐ *S*). In this manner, they represent survivorship assuming that competing events can be treated as censors. The curves in panels representing development are of cumulative hazards transformed according 1‐ *exp*(‐ *S*). They represent the fraction of individuals that made a transition, treating deaths as censors and not as competing events

Transformed cumulative hazard functions per stage allow a comparison of relative magnitudes of mortalities between stages and ages (Figure 3a‐e). These curves can be interpreted as survivorship curves, assuming that competing developmental transitions can be treated as censors. For ages where these curves are steep, mortality rates are high. Where they become flat, mortality becomes negligible. Figure three shows that, by the end of the observation period, the cumulative hazard is lowest for prehatching embryos (Figure 3e) and highest for embryos that remained in the earliest developmental stage (Figure 3a). In stage 1, most deaths occur in the first month. After that, remaining in this stage does not impose an immediate disadvantage in terms of further mortality relative to the other stages. The death rate decreases with age to become zero in most stages and treatment groups. By the age where all surviving embryos are in the rewetting phase, there are hardly any new deaths occurring. Table 2 summarizes which variables were retained in the models for survival per stage. Table S1 does that for a dataset restricted to the observations made before the start of the long interval (days 96–133, Figure 2). There are significant fixed effects for stages two and five, and other effects suggested by Figure 3 were not significant. Embryos that are desiccated in stage 2 have an increased death rate (confidence interval [0.11, 1.49]) and embryos which have previously spent a longer duration in stage 1 have a decreased death rate (slope [−0.12, −0.02]). In stage 5, there is negligible mortality in the wet phase (Figure 3 and Supporting Information Figure S5). Death rates in the prehatching stage are increased by any of the desiccation regimes, even when eggs were in air with 100% relative humidity (increase in mortality rate for H_2_O [0.33, 3.00]). Confidence intervals for mortality rates of KNO_3_ ([2.45, 4.81]) and NH_4_H_2_PO_4_ ([2.51, 4.87]) desiccation regimes overlap with H_2_O, for KCl it does not ([3.68, 6.31]). The order of mortality rates matches the intended strengths of desiccation. In the prehatching stage, mortality increases with temperature ([0.89, 1.81]). The supplementary material contains Table S3 and figures of survivorship curves per stage and per period (Wet/Dry/Rewetted). These results suggest that the lower survival in the driest desiccator was caused by a briefly elevated mortality at the start of the rewetting phase. There are no significant effects of parental age (Table 2). However, in stage 5 and when each period is analyzed separately (Table S3), we recover parental age effects not found when the data are jointly analyzed across periods. Prehatching embryos with older parents have lower mortality in the dry period [parameter estimate −1.49 (*SE* = 0.42)] but a higher mortality in the following rewetted period (1.43 (0.75)) resulting in no net overall effect.

**Table 2:** Survival analysis per stage across periods

Stage	Random effects			Fixed effects				
	Parent	Date	Plate	Salts	Periods	Temp	Age	Preceding
1	55.46 <0.001	71.55 <0.001	12.41 <0.001	NS	NS	NS	NS	
2	NS	NS	NS	NS	5.19 0.023	NS	NS	11.67 <0.001
3	NS	9.08 0.003	NS	NS	NS	NS	NS	NS
4	NS	4.78 0.029	NS	NS	NS	NS	NS	NS
5	NS	NS	NS	157.02 <0.001	NS	37.25 <0.001	NS	NS

Note

"NS" indicates nonsignificant effects. Period × treatment interactions were never significant and are therefore not listed. Significant effects are indicated by *χ* ^2^ statistic and *p*‐value. Parameter estimates of significant effects are presented in the main text.

When we inspect the results on the random effects, variances between parental pairs and between plates are significant in stage 1 only, while random effects of collection date are present in three out of five stages (one, three and four). A linear collection date effect is not different from zero for stage 1 (0.004 (0.004)). Mortality rates decrease with collection date in stages three (−0.052 (0.017)) and four (−0.032 (0.015)), respectively. However, these linear effects are insufficient to explain all variation between collection dates (AIC differences larger than ten).

### 3.2 Development

The transformed cumulative hazard functions for rates of development in Figure 3f–J represent the proportions of embryos that have made a developmental transition as a function of the time already spent in each stage (assuming deaths can be treated as censors). For embryos in stages one and five, the cumulative hazards clearly stop increasing at a certain age within‐stage before all individuals have made a developmental transition (Figure 3f,J), showing that diapause I and III occur irrespective of mild desiccation. There is a small fraction of abortive hatchings from stage 5. Diapause II is not observed as a clear levelling off in the fraction that made a transition (Figure 3g,h), even with prolonged but mild desiccation. For stage 2, the cumulative hazard does seem to stop increasing but at a value implying that the fraction remaining in diapause in this stage would be very small, below 5%. We also see a similar amount of individuals that do not make a developmental transition from stage 4 into 5, where no diapause is expected. What we clearly observe is that the curves for development in stage 2, three, and four, all show a period of about twenty days between days 20 and 50 with fewer developmental transitions. When data are censored before the single long time interval between observations, there is no such period. We conclude that there are no slow/fast alternative developmental trajectories in these stages. For the shortened dataset and relative to the control, embryos in saturated air develop significantly more slowly (−0.37 (0.17)) from stage 1 into 2. However, the effect is not clearly visible in graphs, there are no significant effects for the other desiccation levels and the effect is not detected in the complete data or when the dry period is separately analyzed. We therefore denote it as spurious. Desiccation changed the developmental rate from stage 2 into stage 3 relative to wet conditions (Table 3, parameter estimates H_2_O 0.27 (0.23); KNO_3_ –0.21 (0.25); NH_4_H_2_PO_4_ –0.57 (0.26)); KCl 1.51 (1.06)). This pattern of estimates suggests a nonlinear effect of desiccation intensity, but that strongly depends on the estimate for KCl which has large sampling variance and is the least reliable. The pairwise difference from the control group is only significant for the NH_4_H_2_PO_4_ treatment. These differences are not discernible in Figure 3g. However, they are in Supporting Information Figure S7. Individuals that spend more time in stage 1 have a slower development rate from stage 2 into 3 (Table 3, slope −0.030 (0.006)). Supporting Information Figure S9 in the supplement suggests that for developmental rates from stage 4 into 5, KCl and the control groups have a lower rate of development than other treatments. The mixed Cox models confirm this, with development into stage 4 significantly increased relative to control in a desiccator with H_2_O (1.73 (0.41)) KNO_3_ (1.49 (0.43)) and NH_4_H_2_PO_4_ (1.18 (0.46)). The rate of development in stage 4 tends to be accelerated for embryos that have spent more time in the preceding stage (slope 0.044 (0.024), Table 3, Tables S2, S4). The rate of spontaneous hatching from stage 5 is significantly smaller for embryos that are in the desiccators (parameter estimate −2.24 (0.72)), and there are no abortive hatchings in the rewetting phase (Supporting Information Figure S10).

**Table 3:** Analysis of developmental rates per stage, across periods

Stage	Random effects			Fixed effects				
	Parent	Date	Plate	Salts	Periods	Temp	Age	Preceding
1	79.68 <0.001	8.97 0.003	NS	NS	NS	NS	NS	
**2**	NS	8.55 0.003	4.53 0.033	10.34 0.016	NS	NS	NS	30.31 <0.001
3	NS	NS	7.46 0.006	NS	NS	NS	NS	NS
**4**	5.91 0.015	5.04 0.025	6.21 0.013	19.32 0.001	NS	NS	NS	NS
5	NS	NS	NS	NS	11.39 0.003	NS	NS	NS

Note

"NS" indicates nonsignificant effects. Period × treatment interactions were never significant and are therefore not listed. Significant effects are indicated by *χ* ^2^ statistic and *p*‐value. Parameter estimates of significant effects are given in the main text. For stages written in bold, we did not include time intervals in the rewetted period in the analysis.

We find significant variation between pairs for the developmental rates in first and fourth stage (Table 3). Plate variance effects occur in slightly more developmental stages than for survival rates (Tables 3 and Tables S2, S4). The parental pair effect on the rate of development from stage 1 into stage 2 is not correlated with that for development in stage 4, nor with the pair effect on mortality in stage 1. The pair effect on the mortality rate in stage 1 is significantly and negatively correlated with the development rate in stage 4 (Spearman's rank correlation *r*_s_ = −0.63, *p* = 0.03). At the level of pair variation, low mortality in stage 1 is associated with faster development from stage 4 into the prehatching stage.

When we fit linear collection date effects in the mixed effect Cox models, the model AIC always increases while estimated effects differ from zero. In stages one and two development slows down with collection date (−0.013 (0.003); −0.019 (0.005)), in stage 4 it accelerates (0.06 (0.02)).

### 3.3 Multistate overview

**Figure 4:**
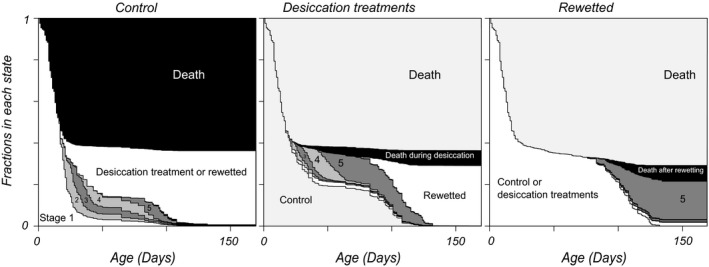
Proportions of embryos in different states (stages of development, death) in dependence on their age. Proportions were estimated using multistate survival analysis. Wet, dry (humid air) and rewetted periods are detailed in separate panels. Each panel only shows the fractions of embryos in the different developmental stages for the focal period. In the left panel, embryos that entered the dry and rewetted regimes are categorized separately as being in that state. In the middle panel, the distribution of embryos in the dry regime over different states (including death) and the fraction that entered the rewetted state are shown. In the right panel, the distribution of embryos over stages in the rewetted regime are detailed

**Figure 5:**
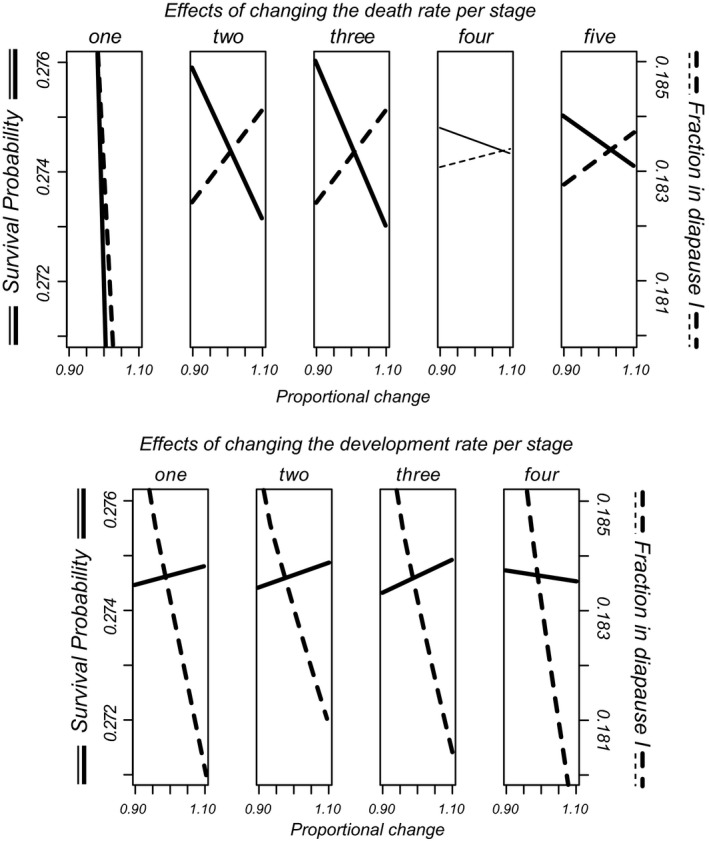
Contributions of different mortality rates and rates of development to expected total number of eggs and to the proportion of individuals in diapause I at the end of the experiment (control conditions). Per panel, a specific rate was changed proportionally (decreases and increases) and resulting values for the two life history components are plotted. In this manner, the plotted lines represent the dependence of the life history components on the rates. Dependencies for which the bootstrap confidence interval for the slope did not include zero are drawn in bold

Figure 4 presents an overview of fractions of embryos in different stages in dependence on age, with different panels for the different regimes. The panels again show the decreases in rates when embryos have been a while in a treatment—the boundaries between states become more horizontal. The amount of events is overall small in the rewetting period. Among the individuals that did not hatch, stage 5 embryos are the most numerous at the end of the experiment, suggesting that in the environments we investigated, diapausing individuals are mainly of diapause type III after some time. Note also that among the rewetted embryos, there is a small fraction of individuals in stage 3. This could not be seen in Figure 3h. The data suggest that after some time, there are small numbers of individuals remaining in stages two to four, and larger ones in the first and last stages. When we plot the dependency of the total number of surviving embryos and the fraction in diapause I on the rates of survival and development (Figure 5), it becomes clear that changes in nearly all rates have effects on these two fitness components but with different strengths. The percentile confidence intervals for the bootstrapped slope estimates did not include zero except for mortality in stage 4. Effects of rates on number surviving and fraction in diapause I are of opposing sign except for the death rate in stage 1. If individuals want to increase the fraction ready for an extra generation within a season, they should increase all developmental rates and decrease mortality rates in stages two, three and five for earlier collection dates and in younger parents. We found such date effects in the rates of development from stages one and two and the opposite effect in the mortality during stage 3.

### 3.4 Distributions across stages

**Figure 6:**
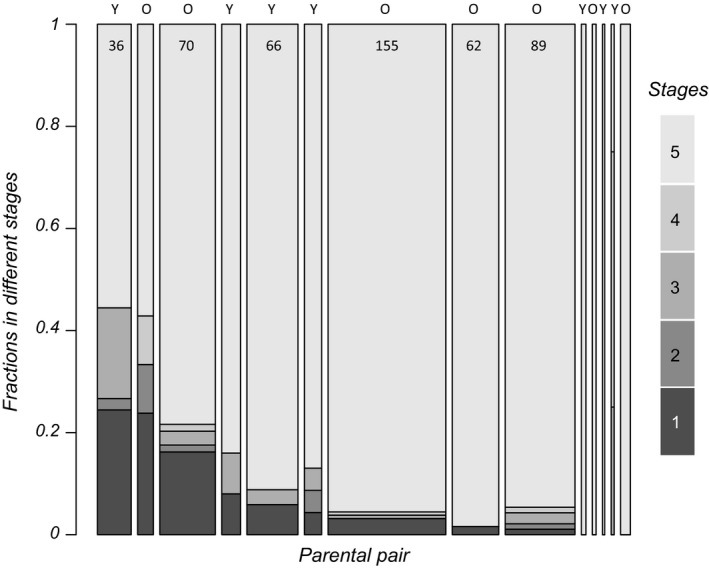
Distributions of embryos over developmental stages (I to V) and parental pairs at rewetting. Widths of bars per pair correspond to sample size (embryos alive at rewetting). Above each bar, it is indicated whether the pair belonged to the group of young (Y) or older (O) pairs. Developmental stages are colored in different shades of gray, with the first developmental stage the darkest. Parental pairs are ordered according the fraction in stage 1, with the largest fraction on the left

**Figure 7:**
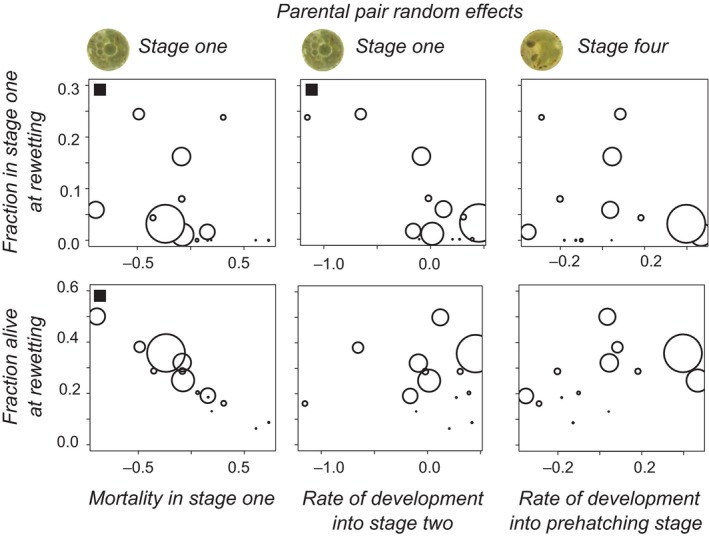
Bubble plots showing the dependence of the fraction of individuals in diapause I and the fraction of individuals surviving at rewetting on random pair (parental) effects on mortality (stage 1) and development (stages one and four). The size of each point is proportional to the number of eggs per parental pair, i.e. the amount of data on which the random effect prediction is based. Black squares indicate pairs of variables for which their Spearman's rank correlation coefficient is significantly different from zero

At first rewetting, 42 individuals were in stage 1, 6 in stage 2, 19 in stage 3, 7 in stage 4, and 525 in stage 5. In 21 out of 67 clutches with more than one embryo alive at this moment, embryos in stages one and five co‐occur, in agreement with expectations for diversified bet‐hedging. We only analyzed fractions in stages one and three relative to stage 5. For the fraction of embryos in stage 1 relative to stage 5 at rewetting, we found significant differences between parental pairs (Figure 6, *χ* ^2^ (13) = 53.19, *p* < 0.001). The fraction in stage 1 at rewetting goes from zero to 24% depending on the parental pair. Parental pair random effects on the fraction in stage 1 at rewetting correlate negatively with the mortality random effect in stage 1 (Spearman's rank *r_s_* = −0.61, *p* = 0.021, Figure 7). There is a negative correlation (*r_s_* = −0.55, *p* = 0.041) with the speed of development from stage 1 into 2. These outcomes are in agreement with expectations based on Figure 5. When we correlate the fraction of embryos alive at rewetting per pair with the significant parental pair random effects, we find that it correlates negatively with the mortality effect in stage 1 (*r_s_* = −0.97, *p* < 0.001, Figure 7).

The fraction of embryos that are in stage 3 at rewetting differs between parental pairs. It is smaller for older parents (effect estimate −2.06 (SE = 0.53); *χ* ^2^ (1) = 17.8, *p* < 0.001). For the distributions over stages at the end of the observation period, we find the same effects. These results suggest different speeds within treatments at which the individuals settle in diapauses I and III, but no consistent treatment differences in the eventual fractions. When we represent the distribution over stages at first rewetting in a graph and label parents according age class (Figure 6), it can be seen that the older parents contributed more individuals overall and in particular in the prehatching stage, as they produced larger clutch sizes sooner. There are no further effects of collection date. At the end of the period where we observed individual state regularly, 139 of the stage 5 individuals had hatched or died.

### 3.5 Hatching

In 37 out of 65 clutches with more than one embryo in stage 5, there were embryos that hatched and others that did not. On average, the hatching probability of the control group where embryos were in water at all times is 12%. For the H_2_O group, it is 27%, in the KNO_3_ desiccator the hatching probability is 13%, from NH_4_H_2_PO_4_ 12% and there was no hatching in the KCl desiccators. Mixed models for hatching probabilities did not converge well, and we refitted them as glm's. For the first rewetting, we found significant effects of collection date (*χ* ^2^ (11) = 62.90, *p* < 0.001), a desiccation treatment effect (*χ* ^2^ (5) = 60.04, *p* < 0.001), and an effect of hatching date (*χ* ^2^ (3) = 29.99, *p* < 0.001). In the H_2_O treatment, the intercept term is increased relative to the control (H_2_O 2.92 (0.46)). There were no significant correlations of parental pair random effects for survival or development with hatching probability per female. When the categorical collection date effect was replaced by a linear effect of collection date, this did not suffice to capture all variation between dates. Moreover, hatching probability increases with collection date (0.037 ; *SE* = 0.011): eggs from later clutches hatch with a larger probability.

When we inspected embryos before the second rewetting (Table 4), a fraction of the eggs had developed further starting from stages one to four. Other embryos had remained in the stages where they were two months before (stages 1, 2, 3, and 5). Survival did not differ significantly between stages. Correlations between parental effects on developmental rate and mortality in stage 1 and the fraction of individuals in stage 1 were slightly weaker (respectively *r_s_* = −0.42 and *r_s_* = −0.45) and nonsignificant. Overall, survival was 83% across this period. When peat water was added, 48% of the stage 5 individuals hatched. We found significant effects of storage temperature (χ^2^ (1) = 4.05, *p* = 0.04) and of the date where the embryos were rewetted before (*χ* ^2^ (3) = 26.84 *p* < 0.0001). For embryos stored at 20.5 ºC, the hatching probability was reduced (parameter estimate effect −0.53 (0.27)). Two first rewetting dates had significantly reduced hatching probabilities at the second rewetting (parameter differences approx. −1.5). There were again no pair effects on hatching probability.

**Table 4:** Developmental stages at second rewetting. A two month‐period separated the follow‐up of embryos after a first rewetting from the second rewetting. Numbers of embryos are given for each combination of states at the end of the follow‐up period and at the moment of rewetting

	Stage at second rewetting					
	1	2	3	4	5	Dead
State at end of follow‐up after first rewetting						
1 before embryogenesis	**21**	1	1	0	5	11
2 somites	0	**3**	0	0	1	1
3 head formed	0	0	**8**	1	7	3
4 pigmented	0	0	0	**0**	3	2
5 prehatching	0	0	0	0	**309**	79

Note

Values in bold denote embryos that remained in the same stage.

### 3.6 Coiling

We estimated rates of change of the tail coiling orientation with a glm as in Harney et al. (2013). In stage 4, we found a significant treatment × time in stage interaction (χ^2^ (4) = 908.84, *p* < 0.0001) and differences between parental pairs (χ^2^ (6) = 13.59, *p* = 0.035), but none of the parameter estimates were significantly different from zero. When simplifying the model to a single effect of time within age, we found that it was nonsignificant.

In stage 5, there is a significant treatment × age within stage interaction (χ^2^ (3) = 9.072, *p* = 0.0284), but again no separate parameters could be shown to be significantly different from zero. For the group desiccated with NH_4_H_2_PO_4_, the rate tends to be lower (−1.48 (0.82), *p* = 0.07). Simplifying to a single covariate—age within stage 5—we find that the probability of changing direction decreases with age in stage 5 (parameter estimate (−0.092 (0.038)). According to this simple model, the probability to change coiling direction at age zero within stage 5 is 5% per day and at day forty 0.1%. For stage 4, the probability to change coiling direction is 7% per day.

## 4 DISCUSSION

We have estimated rates of survival and development in five embryonal stages in the annual killifish *Austrolebias bellottii*, assessed factors affecting their variability and investigated how rates affect life history fitness components.

Irrespective of whether environmental switches were imposed, a general slowdown in rates of death and development occurred when the environment remained constant for a prolonged time. Desiccation plasticity seems relatively limited. Desiccation had effects on rates of hatching shortly after treatment but these effects were not lasting. Pair variation in the rates of development and survival in the first developmental stage contributes to the proportion in that stage at the moment of rewetting and pair variation in the death rate in stage 1 affects the total number of embryos alive, two to three months later.

### 4.1 Adaptive development

We can assess three aspects of adaptation in the development of annual killifish using results from this experiment. First, we can check whether patterns of individual variation are consistent with bet‐hedging variation. Second, we can inspect parental age and collection date effects to check whether development might be adapted to exploit opportunities to complete an additional generation within the same year. Third, we can check whether desiccation could be used as a cue for seasonal timing, not only announcing the arrival of the dry season, but also the rewetting when it ends.

We found co‐occurring fractions of individuals in diapause I and III in many clutches. Many parental pairs and many clutches have fractions of embryos in different developmental stages at rewetting, consistent with diversified bet‐hedging. Patterns of exit from diapause characterize germ banking strategies (Evans & Dennehy, 2005). Here, hatching is probabilistic, also at the within‐clutch level. The fraction of individuals in diapause I at rewetting, a key aspect of the delaying strategy, is not plastic and depends on parental pair variation. This leaves the possibility open for maternal determination of this strategy, as in *Nothobranchius* (Polačik et al., 2017). We could not find any coupling of rates of development to hatching probabilities. Processes of diapause entry and exit seem different.

Evidence for adaptive responses to exploit opportunities for an extra generation is ambiguous and relatively weak. Younger parents are not producing faster‐developing embryos. Development in late developmental stages accelerates with collection date. Hatching probabilities increased substantially at the second rewetting. These effects are unexpected according to this hypothesis. Development in early stages decelerates with collection date, in agreement with this hypothesis. At occasions very late in the season preference should be given to remain in the egg bank longer. The positive collection date effect on hatching probability is in agreement with embryos timing seasonal progress using their own individual age. Younger embryos then assume that they are early in the year.

Desiccation seems to announce the dry season relatively well: In agreement with this hypothesis, late development is accelerated in the presence of mild desiccation and so is the hatching response. The collection date effect on late development could also be explained as a response for a cue that seasons progress and that a dry period will be followed by rewetting. The collection date effect on hatching disagrees with this hypothesis.

We have found stage‐specific effects of collection date (stages three and four) and of the desiccation treatment (prehatching stage) on mortality. If such effects occur in the field, then they will affect which decisions taken by individuals are optimal. Altogether, responses to age, date and desiccation seem to indicate that a tool such as dynamical programming will be needed to assess adaptation in development and hatching well and that we need to move beyond hypotheses that we can assume and test without detailed modeling and ignoring stage‐specific survival. Similarly, a solid demonstration that the distribution over diapauses I and III and probabilistic hatching represent bet‐hedging will require a demographic model and fitness calculations of the entire life cycle (Childs et al., 2010; de Jong, Haccou, & Kuipers, 2011).

Important is that we could demonstrate by means of simulations and our data that random pair variation found in rates during development is "visible" for selection: rates of development and survival affect components of fitness. If adapted, they are likely kept at their present values because of trade‐offs between rates and fitness components or because selection on the life history components we investigated is weak.

### 4.2 Annual versus nonannual rivulids

In comparison to nonannual rivulids where initially over 10% of individuals changed coiling per day (Varela‐Lasheras & Van Dooren, 2014), the activity of prehatching annual fish embryos seems overall lower from the start. Overall, the effects of desiccation are limited and different from the pattern observed among nonannual rivulids. The desiccation regimes in this study are much prolonged in comparison to Varela‐Lasheras and Van Dooren (2014), where the median duration was eight days, with visible effects on the state of the eggs after one hour. There, delays in response to brief desiccation were early in development and desiccation provoked an increased incidence of delayed hatching (similar or equal to diapause III) in stage 5 in some species. Regarding early development in the first stage of the Argentinean pearlfish, we only found a significant desiccation effect in a reduced dataset and for the weakest desiccation level, casting doubt on its existence. The speed of development near head formation was slowed after rewetting for one desiccation level and showed a positive response to desiccation late in development. In nonannuals, desiccation significantly increased mortality in stages three and four and decreased in *Cynodonichthys magdalenae* again after return to water. In the annual species, developing embryos are quite resistant to the desiccation levels we applied as survival effects are limited to the prehatching stage.

Slowing down during early development in nonannuals in response to desiccation might be mostly an immediate response, therefore quiescence, even while there are some lagged effects (however, see Furness et al., 2018). If genetic assimilation of diapause has occurred in killifish via the evolution of plasticity in response to desiccation, then there has clearly been further plasticity evolution after the emergence of diapause‐like characteristics. The plasticity now involves an accelerating effect.

### 4.3 The three diapauses

According to Wourms (1972c), diapause I is common in South American *Austrolebias* killifish, whereas diapause III is obligatory and diapause II facultative, as in *Nothobranchius* (Furness, Lee, et al., 2015; Wourms, 1972c). In agreement with that, diapause I and III occurred in benign conditions, consistent with a requirement for diapause that can occur in the absence of an environmental stressor. Note that there were no escape embryos in the sense of Polačik et al. (2017) that reach the prehatching stage within a month.

If diapause II occurred in this experiment, it did so in below 5% of the individuals that stayed alive in stage 2 and after more than a month in that stage. Similar proportions of individuals remained in the fourth developmental stage, where no diapause has ever been inferred. We also observed embryos that spent two months in stages two or three between the first and second rewetting.

Recently, diapause II was detected in *Austrolebias nigrofasciatus* (da Fonseca et al., 2018). Its incidence differed depending on incubation conditions. Lowered oxygen levels might be an environmental cue occurring in the closed vials with batches of eggs used by da Fonseca et al. (2018), or when organic matter present decomposes. Diapause II might have more characteristics of a quiescence than a diapause, occurring mostly in conditions of environmental stress. It might occur rarely in *Austrolebias* in comparison to the other diapauses, but data for most species are currently lacking. Diapause II is not constitutive for this genus and our results on the stage where diapause II occurs can be more parsimoniously explained by the general decrease of rates in all stages.

Several papers recently brought new data and ideas on the diapauses in annual fish. Furness (2016) believes diapause I to have a short duration in benign conditions and to be mainly brought about by harsh conditions. Diapause II, after the phylotypic stage, would contribute most to variability in developmental duration (Furness, 2016). Our results contradict this. Podrabsky et al. (2001) found that *Austrofundulus* embryos resisted desiccation best in diapause II. We found that most developmental stages suffer no survival effects of prolonged mild desiccation in *Austrolebias*. Given the results here, diapause II is certainly not the most prominent stage of arrest in all annual fish as proposed by Furness, Reznick, et al. (2015). Being more facultative than diapause III at least in *Austrolebias*, it cannot be equated to the annualism syndrome.

It has not been clear which criteria were previously followed to assign embryos to diapause or direct‐developing pathways (Furness, Lee, et al., 2015; Furness, Reznick, et al., 2015) and there has been some circularity in the data analysis, with developmental outcomes used as an explanatory variable of the same process. Data on *Austrolebias nigripinnis* in Furness, Reznick, et al. (2015) suggest that developmental trajectories are not very dichotomous between embryos with direct development to a prehatching stage or not, whereas direct developers in *Nothobranchius* have significantly larger heads. This suggests that next to developmental delays, morphological embryonal life history traits have evolved differently in the different annual groups.

### 4.4 Parental effects

We found a very limited number of significant effects of parental age on the survival and development of embryos and hatching. However, we did find variation between parental pairs for survival and developmental rates, which could encompass genotypic variation. Several of the pair effects on rates could be correlated to fitness components, and two random effects were correlated as well. We did not find parental pair effects on hatching and therefore on the exit from diapause. The potential for evolutionary responses in this system seem larger with respect to the composition of the germ bank than in the exit from "germ banking".

## 5 LIMITATIONS

Our incubation protocol differed from incubation environments used by others, that is,with eggs placed either on top of or under a small layer of peat moss. Alternative setups to study desiccation effects might be relevant for annual fish, but care should be taken in controlling environmental variability between eggs. In the field, it is expected that changes in soil dryness will make availability of oxygen and dryness covary, such that an experiment designed to separate oxygen and desiccation effects would be crucial to explore plasticity and survival effects of both. In an experiment where a range of durations of desiccation is imposed, a direct comparison of annuals and nonannuals would be possible, as the environmental design would include environments allowing survival and development for either group.

Our data loggers did not allow us to demonstrate that we reached levels of desiccation precisely as intended, and there might be other side effects of salts than just controlling humidity levels. The desiccators with different salts clearly smelled differently and it can be expected that molecules enter the medium with embryos as well. If such effects were there, they seem to have been limited.

We reserved the term diapause for cohorts with an absence of events for a prolonged period, as we did not collect accessory individual data on their metabolism or on the advancement of morphological structures which could have provided measures of diapause depth at the individual level. We did record the changes in coiling direction of individual embryos, finding that it occurred at a lower rate than in nonannuals. Future studies should try to correlate different invasive and noninvasive methods of measuring metabolic activity, to see which noninvasive measures can be used as fast and reliable proxies across a wide range of killifish species in the future.

## 6 CONCLUSIONS

Mild desiccation and rewetting affect survival, rates of development and hatching probability in *Austrolebias bellottii*, but not the fractions of embryos that arrest development in particular stages. We found individual variability in agreement with diversified bet‐hedging. The incidences of diapause have become relatively independent of the occurrence of mild desiccation, as if they have become assimilated. In contrast to the responses observed in nonannual rivulids, *Austrolebias* accelerates development into the prehatching stage in response to mild desiccation. Rates of development and survival during development are not phenotypically neutral; they can have effects on fitness components and be visible for natural selection. Plasticity and date effects suggest that that individuals might predict the start of the dry season and that the developmental system is not consistently adapted to exploit opportunities for an extra generation within the same year.

## CONFLICT OF INTEREST

None declared

## AUTHOR CONTRIBUTIONS

TJMVD conceived the study. IVL and TJMVD performed the experiments, analyzed the data, and wrote the manuscript.

## DATA ACCESSIBILITY

The raw data and R scripts are archived on Dryad Digital Repository: https://doi.org/10.5061/dryad.8540192
