# Supplementary material for: Embryonal life histories: Desiccation plasticity and diapause in the Argentinean pearlfish Austrolebias bellottii
Source: Ecol Evol. 2018 Oct 30;8(22):11246–60. doi: 10.1002/ece3.4599 (PMC6262906; doi:10.1002/ece3.4599)
Supplement: Supplementary file 1 [file ECE3-8-11246-s001.docx]

Desiccation plasticity and diapause in the Argentinian pearlfish *Austrolebias bellottii*

Tom JM Van Dooren and Irma Varela-Lasheras

Supplementary Material

Table S1. Survival analysis per stage censored before the long interval between observations. "NS" indicates non-significant effects. Period × treatment interactions were never significant and therefore not listed. Significant effects are indicated by the *χ*^2^-squared statistic and *p*-value. There were no desiccation levels which had significantly different parameter estimates from desiccators with 100% relative humidity. Period effects could not be fitted in most models.

| Stage | Parent | Date | Plate | KCl | KNO_3_ | NH_4_H_2_PO_4_ | Salts | Temp | Age | Preceding |
| --- | --- | --- | --- | --- | --- | --- | --- | --- | --- | --- |
| 1 | 54.47  < 0.001 | 70.68  < 0.001 | 10.10  0.001 |  |  |  | NS | NS | NS |  |
| 2 | NS | 5.08  0.024 | NS |  |  |  | NS | NS | NS | 8.51  0.004 |
| 3 | NS | NS | NS |  |  |  | NS | NS | NS | NS |
| 4 | NS | NS | NS |  |  |  | NS | NS | NS | NS |
| 5 | NS | NS | NS |  |  |  | NS | NS | NS | NS |

Table S2. Analysis of developmental rates per stage, censored before the long interval. "NS" indicates non-significant effects. Period × treatment interactions were never significant and therefore not listed. Significant effects are indicated by the *χ*^2^-squared statistic and *p*-value. There were no desiccation levels which had significantly different parameter estimates from desiccators with 100% relative humidity. For stages written in bold, we did not include time intervals in the rewetted period in the analysis.

| Stage | Parent | Date | Plate | KCl | KNO_3_ | NH_4_H_2_PO_4_ | Salts | Temp | Age | Preceding |
| --- | --- | --- | --- | --- | --- | --- | --- | --- | --- | --- |
| 1 | 76.09  < 0.001 | 9.38  0.002 | NS |  |  |  | 13.98  0.007 | NS | NS |  |
| **2** | NS | NS | 7.81  0.005 |  |  |  | 17.52  < 0.001 | 5.36  0.021 | NS | NS |
| 3 | NS | NS | NS |  |  |  | NS | NS | NS | NS |
| 4 | NS | 38.52  <0.001 | NS |  |  |  | NS | NS | NS | 8.92  0.003 |
| 5 | NS | NS | NS |  |  |  | NS | NS | NS | NS |

Table S3. Survival analysis, per stage and period separately. Significant effects on mortality rates are listed, based on likelihood ratio *χ*^2^ tests. Significant effects are indicated by *p*-values. When the effect was not tested, cells are left empty. For the salts used, those which had significantly different parameter estimates from desiccators with 100 % relative humidity are indicated by black cells. For such salt × stage combinations, pluses or minuses show the sign of the difference in mortality relative to incubation in 100% relative humidity for that stage.

| Stage | Parent | Date | Plate | KCl | KNO_3_ | NH_4_H_2_PO_4_ | Salts | Temp | Age | Preceding |
| --- | --- | --- | --- | --- | --- | --- | --- | --- | --- | --- |
| 1 Wet | < 0.001 | < 0.001 | 0.001 |  |  |  |  | NS | NS |  |
| 1 Dry | NS | NS | 0.043 |  |  |  | NS | NS | NS |  |
| 1 Rewetted |  |  |  |  |  |  | NS | NS | NS |  |
| 2 Wet | NS | 0.016 | NS |  |  |  |  | NS | NS | 0.001 |
| 2 Dry | NS | NS | NS |  |  |  | NS | NS | 0.004 | 0.021 |
| 2 Rewetted |  |  |  |  |  |  | NS | NS | NS | NS |
| 3 Wet | NS | NS | NS |  |  |  |  | NS | NS | NS |
| 3 Dry | NS | NS | NS |  |  |  | NS | NS | NS | NS |
| 3 Rewetted |  |  |  |  |  |  | NS | NS | NS | NS |
| 4 Wet | NS | NS | NS |  |  |  |  | NS | NS | NS |
| 4 Dry | NS | NS | NS |  |  |  | NS | NS | NS | NS |
| 4 Rewetted |  |  |  |  |  |  | NS | NS | NS | NS |
| 5 Wet | No events | | | | | | | | | |
| 5 Dry |  |  |  |  | **+** | **+** | < 0.001 | 0.002 | 0.001 | NS |
| 5 Rewetted | NS | 0.023 | NS | **+** |  |  | < 0.001 | < 0.001 | 0.022 | NS |

Table S4. Development per period and per stage. Significant effects are indicated by *p*-values. When the effect was not tested, cells are left empty. There were no desiccation levels with development rates that were significantly different from incubation in 100% relative humidity for that stage.

| Stage | Parent | Date | Plate | KCl | KNO_3_ | NH_4_H_2_PO_4_ | Salts | Temp | Age | Preceding |
| --- | --- | --- | --- | --- | --- | --- | --- | --- | --- | --- |
| 1 Wet | < 0.001 | 0.006 | NS |  |  |  |  | NS | NS |  |
| 1 Dry | 0.004 | 0.001 | 0.027 |  |  |  | NS | NS | NS |  |
| 1 Rewetted |  |  |  |  |  |  | NS | NS | NS |  |
| 2 Wet | NS | 0.001 | 0.033 |  |  |  |  | NS | NS | 0.003 |
| 2 Dry | NS | NS | NS |  |  |  | NS | NS | NS | 0.001 |
| 2 Rewetted |  |  |  |  |  |  | NS | NS | NS | NS |
| 3 Wet | NS | NS | NS |  |  |  |  | NS | NS | NS |
| 3 Dry | NS | NS | 0.036 |  |  |  | NS | NS | NS | NS |
| 3 Rewetted |  |  |  |  |  |  | NS | NS | NS | NS |
| 4 Wet | NS | 0.032 | NS |  |  |  |  | 0.027 | NS | < 0.001 |
| 4 Dry | 0.016 | < 0.001 | NS |  |  |  | 0.010 | NS | NS | 0.024 |
| 4 Rewetted |  |  |  |  |  |  | NS | NS | NS | NS |
| 5 Wet | NS | NS | NS |  |  |  |  | NS | NS | NS |
| 5 Dry | NS | NS | NS |  |  |  | NS | NS | NS | NS |
| 5 Rewetted | No events | | | | | | | | | |

Fig S1. Transformed mortality cumulative hazards for stage one in different periods. From left to right: before desiccation, during desiccation, and when water was added again. In the two rightmost panels age is rescaled to the time where the regime started (i.e. start of dry period, rewetted). The control group is added to each panel as well, with the average age at which the regime started substracted from the actual age of embryos. This was done to facilitate comparisons between treatments. Black, thin: control group; blue, thin: desiccator with H_2_0; red thick: KNO_3_; blue thick: NH_4_H_2_PO_4_; black, thick: KCl. In the leftmost panel, the confidence interval of the cumulative hazard function is drawn.

Fig S2. Transformed mortality cumulative hazards for stage two in different periods. From left to right: before desiccation, during desiccation, and when water was added again. In the two rightmost panels age is rescaled to the time where the regime started (i.e. start of dry period, rewetted). The control group is added to each panel as well, with the average age at which the regime started substracted from the actual age of embryos. This was done to facilitate comparisons between treatments. Black, thin: control group; blue, thin: desiccator with H_2_0; red thick: KNO_3_; blue thick: NH_4_H_2_PO_4_; black, thick: KCl.

Fig S3. Transformed mortality cumulative hazards for stage three in different periods. From left to right: before desiccation, during desiccation, and when water was added again. In the two rightmost panels age is rescaled to the time where the regime started (i.e. start of dry period, rewetted). The control group is added to each panel as well, with the average age at which the regime started substracted from the actual age of embryos. This was done to facilitate comparisons between treatments. Black, thin: control group; blue, thin: desiccator with H_2_0; red thick: KNO_3_; blue thick: NH_4_H_2_PO_4_; black, thick: KCl.

Fig S4. Transformed mortality cumulative hazards for stage four in different periods. From left to right: before desiccation, during desiccation, and when water was added again. In the two rightmost panels age is rescaled to the time where the regime started (i.e. start of dry period, rewetted). The control group is added to each panel as well, with the average age at which the regime started substracted from the actual age of embryos. This was done to facilitate comparisons between treatments. Black, thin: control group; blue, thin: desiccator with H_2_0; red thick: KNO_3_; blue thick: NH_4_H_2_PO_4_; black, thick: KCl.

Fig S5. Transformed mortality cumulative hazards for stage five in different periods. From left to right: before desiccation, during desiccation, and when water was added again. In the two rightmost panels age is rescaled to the time where the regime started (i.e. start of dry period, rewetted). The control group is added to each panel as well, with the average age at which the regime started substracted from the actual age of embryos. This was done to facilitate comparisons between treatments. Black, thin: control group; blue, thin: desiccator with H_2_0; red thick: KNO_3_; blue thick: NH_4_H_2_PO_4_; black, thick: KCl.

Fig S6. Transformed cumulative hazards for development rates from stage one into stage two in different periods in the experiment. On the y-axis is plotted the fraction in the population that made the transition, assuming that deaths can be treated as censors. From left to right: before desiccation, during desiccation, and when water was added again. In the two rightmost panels age-within-stage is rescaled to the time where the regime started (i.e. start of dry period, rewetted). The control group is added to each panel as well, with the average age at which the regime started substracted from the actual age-within-stage of embryos. This was done to facilitate comparisons between treatments. Black, thin: control group; blue, thin: desiccator with H_2_0; red thick: KNO_3_; blue thick: NH_4_H_2_PO_4_; black, thick: KCl.

Fig S7. Transformed cumulative hazards for development rates from stage two into stage three in different periods in the experiment. From left to right: before desiccation, during desiccation, and when water was added again. In the two rightmost panels age-within-stage is rescaled to the time where the regime started (i.e. start of dry period, rewetted). The control group is added to each panel as well, with the average age at which the regime started substracted from the actual age-within-stage of embryos. This was done to facilitate comparisons between treatments. Black, thin: control group; blue, thin: desiccator with H_2_0; red thick: KNO_3_; blue thick: NH_4_H_2_PO_4_; black, thick: KCl.

Fig S8. Transformed cumulative hazards for development rates from stage three into stage four in different periods in the experiment. From left to right: before desiccation, during desiccation, and when water was added again. In the two rightmost panels age-within-stage is rescaled to the time where the regime started (i.e. start of dry period, rewetted). The control group is added to each panel as well, with the average age at which the regime started substracted from the actual age-within-stage of embryos. This was done to facilitate comparisons between treatments. Black, thin: control group; blue, thin: desiccator with H_2_0; red thick: KNO_3_; blue thick: NH_4_H_2_PO_4_; black, thick: KCl.

Fig S9. Transformed cumulative hazards for development rates from stage four into stage five in different periods in the experiment. From left to right: before desiccation, during desiccation, and when water was added again. In the two rightmost panels age-within-stage is rescaled to the time where the regime started (i.e. start of dry period, rewetted). The control group is added to each panel as well, with the average age at which the regime started substracted from the actual age-within-stage of embryos. This was done to facilitate comparisons between treatments. Black, thin: control group; blue, thin: desiccator with H_2_0; red thick: KNO_3_; blue thick: NH_4_H_2_PO_4_; black, thick: KCl.

Fig S10. Transformed cumulative hazards for spontaneous hatching in stage five, in different periods in the experiment. On the y-axis is plotted the fraction in the population that hatched, assuming that deaths can be treated as censors. From left to right: before desiccation, during desiccation, and when water was added again. In the two rightmost panels age-within-stage is rescaled to the time where the regime started (i.e. start of dry period, rewetted). The control group is added to each panel as well, with the average age at which the regime started substracted from the actual age-within-stage of embryos. This was done to facilitate comparisons between treatments. Black, thin: control group; blue, thin: desiccator with H_2_0; red thick: KNO_3_; blue thick: NH_4_H_2_PO_4_; black, thick: KCl.
